# Inclusive pedagogy in online simulation-based learning in undergraduate nursing education: A scoping review protocol

**DOI:** 10.12688/hrbopenres.13557.1

**Published:** 2022-05-16

**Authors:** Lisa Langan, Phil Halligan, Kate Frazer, Andrew Darley, Lizbeth Goodman, Catherine Redmond

**Affiliations:** 1 MTU Department of Nursing and Healthcare Sciences, Munster Technological University, Tralee, Co. Kerry, V92 HD4V, Ireland; 2 UCD School of Nursing, Midwifery and Health Systems, University College Dublin, Belfield, Dublin 4, D04 C7X2, Ireland; 3 UCD School of Medicine, University College Dublin, Belfield, Dublin 4, D04 C7X2, Ireland; 4 UCD School of Mechanical and Materials Engineering, University College Dublin, Richview Newstead Belfield, Dublin 4, D04 V1W8, Ireland

**Keywords:** inclusive pedagogy, undergraduate nursing student, nurse educator, online simulation-based learning

## Abstract

**Background:** Education is recognised as a constitutional right, however, access to and participation in higher education can be challenging for some students. This has led to the development of various international and local initiatives promoting inclusion, which has increased student representation from marginalised groups. In order to support growing student diversity, teaching and learning (T&L) strategies must encompass inclusive pedagogical principles. Technological advancements have improved opportunities for online T&L strategies and these are becoming an integral component of curricula in undergraduate nursing programmes. Online simulation-based learning (SBL) has gathered momentum in nursing education over the past twenty years. However, it is unclear from the evidence-base how inclusive this educational approach is, and how it can best support the growing diversity among nursing students. This paper outlines the protocol for a scoping review that aims to systematically and comprehensively map the available published and unpublished literature on inclusive pedagogy in online SBL in undergraduate nursing education.

**Methods:** The Preferred Reporting Items for Systematic Reviews and Meta-analyses Extension for systematic review protocols (PRISMA-P) guided this protocol. Arksey and O'Malley (2015) six-stage methodology framework, the Joanna Briggs Institute (JBI) guidelines (2005) and the PRISMA extension for scoping reviews (PRISMA-ScR) will guide the proposed scoping review.

**Conclusion:** It is envisaged that this scoping review will give a broad overview of the evidence for inclusive pedagogy in online SBL at this point. The findings of this review will be used to inform future policy and the pedagogical and technological design of online SBL activities and assist nurse educators to meet the current requirement of inclusive practice.

## Introduction

Education is recognised as a human and constitutional right ( UNESCO, 1960; UNESCO, 2021; United Nations, 1948). However, access to and participation in higher education (HE) can be challenging for some students. Several factors (gender, marital status, pregnancy, race, religion and sexual orientation) are known to create challenges for students ( European University Association, 2021; European Commission, 2020a; European Commission, 2020b). Further, some population groups are identified as being underrepresented in HE (low-income groups, women, minority groups linked to specific characteristics (e.g., ethnic/linguistic/religious/cultural/age or residence, and individuals with disabilities) ( Salmi, 2020). This has led to the promotion of inclusion in education, with a particular focus on marginalised groups in society.

Inclusion can be conceptualised in many ways depending on various standpoints as it is considered highly context and philosophically bound ( Messiou, 2017; Nilholm, 2021). As yet, no universal definition of inclusion exists. In education, the concept of inclusion is influenced by the UN Salamanca Statement regarding special needs education for children ( UNESCO, 1994). That statement highlighted the importance of learner-centredness, the embracing of diversity, inclusion of all and pedagogy, which are equally applicable to the HE sector ( Carballo *et al.*, 2021; Floretta, 2021). The influence of ethical values such as social solidarity and respect towards the diversity of others are also prominent in the inclusion literature ( Nilholm, 2021; Stentiford & Koutsouris, 2021).

Some inclusion terms are used interchangeably in HE literature and need clarification. *Inclusive education* works to identify and remove all barriers to education, and cover everything from curricula to pedagogy and teaching ( UNESCO, 2021). In contrast, *inclusive practice* refers to any specific teaching practices that promote learning and engagement of all students ( Moriña, 2020). *Inclusive pedagogy* does not exclusively focus on teaching practices that educators engage in, but also the how and why ( Florian & Black-Hawkins, 2011). As this concept offers a comprehensive depiction of all the major facets of inclusion, and specifically targets inclusion in HE, it will be the focus of this review.

Moriña (2020), following a systematic review, believes that inclusive pedagogy encompasses four components: *beliefs, knowledge, design and actions*. Beliefs that students can contribute to the learning environment and beliefs around how differences in learning needs are perceived play a pivotal role in how educators develop their inclusive approaches. This can vary from providing for 1) needs common to all or 2) needs specific to sub-groups (e.g., an identified minority group) or 3) individual needs ( Stentiford & Koutsouris, 2021).

Moriña (2020) also outlined the essential inclusive pedagogical knowledge required by educators, in addition to their subject-specific knowledge. Educators need to be informed in the areas of: disability and special needs, of adult learning principles and strategies, of where to go for help when needed; of how to identify and assess difficulties and the legal context of education. Florian and Black-Hawkins (2011) go further and note that the “craft of knowledge” required by educators must also include the iterative process of reflection and problem solving as these shape what is carried out in teaching practice.

The third component “inclusive design” refers to the design of a pedagogy that recognizes the value of all students, involves them in the design of their studies from the beginning and meets the needs of as many students as possible ( Gale & Mills, 2013; Moriña, 2020). An equity/equality, diversity and inclusion (EDI) theoretical framework underpins inclusive design and considers accessibility and participation to the widest extent feasible ( Eikhaug & Gheerawo, 2021). Zhang *et al.* (2017:4) define inclusive design as: “ *the methodology of co-designing technological solutions with user groups to personalise their learning …to best support each individual*”. The interpretation is that regular discourse through co-design with students, creates opportunities for the continuous adaptation of teaching and learning (T&L) strategies. This framework can thus be used to gain insight into real challenges for students and identify shortfalls in inclusion ( Eikhaug, 2019). An example of an inclusive design framework is Universal Design for Learning, where the educator designs teaching strategies with multiple means of representation (the ‘what’ of learning), expression (the ‘how’ of learning) and engagement (the ‘why’ of learning) to cover the needs of all students ( CAST, 2018).

The final element identified by Moriña (2020) refers to the actions or practices that ‘work with’ rather than ‘act on’ students. Developed practices include: flexible learning, student-centred learning and peer learning - the sharing of beliefs, knowledge, and experiences among students ( Aguirre *et al.*, 2020; Aguilar *et al.*, 2020).

Internationally, several reports paved the way forward to promote inclusion for all students in education ( UN, 1989; UN, 2006; UNESCO, 1994; UNESCO, 2017). These have inspired nations in the promotion of EDI in practice ( European Commission, 2020b). In Ireland, six underrepresented priority groups in HE were identified ( HEA, 2018) and initiatives such as the Delivering Equality of Opportunity in Schools (DEIS) programme, the *Disability Access Route to Education* (DARE) initiative, the *Programme for Access to Higher Education* (PATH), and the *Higher Education Access Route* (HEAR) were developed ( Nic Fhlannchadha, 2018). This has resulted in a substantial increase in enrolment of these target groups into HE and a rapid increase in the diversity of our student population ( IUA, 2018; HEA, 2020). This scene is replicated across Europe and the developed world and has implications for educators. They must now rethink traditional T&L strategies and pedagogies, to encompass inclusion principles and plan for current and future increases in student diversity ( Arellanes & Hendricks, 2021; Neilis *et al.*, 2021).

## Teaching and learning in nursing education

In tandem with the EDI initiatives, educators are also influenced by the many technological advancements that have occurred recently in academic settings and nursing is no exception. T&L strategies are becoming more portable and digitally-based ( Garcia-Tudela *et al.*, 2020) and include a range of simulation-based learning (SBL) pedagogies including online simulations, virtual patients, serious gaming and 3-D simulations ( Asad *et al.*, 2021; Maheu-Cadotte *et al.*, 2021). SBL is an educational method guided by a theoretical foundation used to “ *replace or amplify real experience with guided experiences*” ( Gaba, 2004, p.i2) and improve nursing students’ knowledge, communication and critical thinking skills as well as technical skills ( Dubovi, 2018; Linn *et al.*, 2019). The rapid growth of online simulation in nursing education reflects the advantages of this pedagogy: it is less time intensive than face-to-face SBL, it does not require significant resources (for example, high fidelity mannequins and facilitators) to function, and it has been shown to be comparable or superior to other simulation methods ( Borg Sapiano *et al.*, 2018; Dubovi, 2018). Other advantages include its flexibility and ease of access, it ensures standardisation of approaches to facilitation and debriefing for all, it allows repetitive practice and can be utilised simultaneously by all ( Havola *et al.*, 2021; Tinôco *et al.*, 2021).

While online SBL does promote inclusion for all students by introducing a flexible and accessible learning strategy where students can learn at their own pace and at their chosen time and place, many of the underpinning theories supporting SBL were not developed to specifically target EDI principles. In order for online SBL to be a fully inclusive pedagogy an EDI theoretical framework should also be used to guide the process of the design of the simulation ( Dede & Richards, 2020; Eikhaug & Gheerawo, 2021). Such frameworks are available, but it is unknown if nurse educators purposely utilise an EDI theoretical framework as a guide.

In summary, as online SBL is becoming more prevalent in undergraduate nursing education it is important to anchor this pedagogy to principles that support student diversity and inclusion. However, an initial exploratory search of the literature showed a lack of clarity regarding if and how inclusive pedagogy is being incorporated into online SBL. A comprehensive approach to inclusion would include all four components of inclusive pedagogy; however to date no primary research study has explored this. Therefore, a systematic synthesis of the literature is necessary to identify the degree to which each of the components of inclusive pedagogy has been reported and highlight barriers and facilitators to inclusion. This will enable us to address gaps in the literature as well as to make suggestions and recommendations for policy and practice. Furthermore, the findings from the scoping review will be used to reflect on how emerging trends may impact nurse educators' delivery of and nursing students experience of inclusive pedagogy.

## Protocol

This scoping review will provide the rigorous mapping of the body of literature, to identify the presence of inclusive pedagogy in online SBL in undergraduate nursing education and will aim to identify any existing gaps in the literature ( Pham *et al.*, 2014; Munn *et al.*, 2018). Arksey and O'Malley’s (2005) six-stage methodology framework, the Joanna Briggs Institute (JBI) guidelines (2015) and the Preferred Reporting Items for Systematic Reviews and Meta-analyses for systematic review protocols (PRISMA-P) checklist has guided this scoping review protocol ( Moher *et al.*, 2015) (see Langan *et al.*, 2022); and the PRISMA-ScR will guide the subsequent review ( Page *et al.*, 2021; Tricco *et al.*, 2018).

## Design

### Stage 1: Identification of the scoping review research question

The PCC mnemonic (Population, Concept and Context) was used to create the research question ( Peters *et al.*, 2020). In this instance, the population identified are undergraduate nursing students and nurse educators; the concept is inclusive pedagogy, and the context is online simulation-based learning in undergraduate nursing education (see Table 1). Following the initial search of the literature and iterative discussion with the author team, the primary research question that will guide the review is:

***What is known regarding inclusive pedagogy in online simulation-based learning among nurse educators and undergraduate nursing students?***

**Table 1.** PCC and Search Strategy.

PCC	Search String
**Population/Participants-** Undergraduate nursing students involved in online SBL in nursing education and nursing educators of undergraduate nursing students	(educator* OR instructor* OR lecturer* OR teacher* OR tutor* OR professor* OR trainer* OR preceptor* OR academic* OR coach* OR undergraduate* OR freshman* OR sophomore* OR undergrad* OR underclassman OR upperclassman OR student* OR co-ed OR novice* OR trainee* OR participant* OR scholar* OR learner* OR pre-reg* OR "pre licensure" OR baccalaureate OR fresher* OR tutee AND (nurs*)
**Concept:** Inclusive pedagogy	“inclusive pedagogy”
	(value OR merit* OR worth OR principle* OR ethic* OR moral* OR standard* AND inclus*
	(belief* OR acceptance OR bias OR assumption OR attitude* OR confidence OR expectation* OR judgement* OR opinion* OR theory OR thinking OR respect OR understanding* OR hypothesis OR impression* OR intuition OR presumption* AND inclus*
	(knowledge OR abilit* OR expertise OR insight* OR philosophy OR proficiency OR comprehension) AND inclus*
	(inclusive OR “inclus* theory” OR universal OR participat* OR good OR UX OR “user experience”) AND design
	codesign OR co-design
	inclusion OR UDI OR UDL OR “design for all” OR accessibility OR adaptability OR flexibility OR learner-cent* OR involvement OR “active participation” OR EDI OR equality OR equity OR diversity
	(action OR activity OR process OR steps OR practice) AND inclus*
**Context:** Online SBL in undergraduate nursing education	“nurse education” OR education OR learning OR scholarship OR study OR teaching OR training OR pedagogy OR tuition OR instruction OR coaching OR practise OR practice OR curriculum OR curricula OR “problem based learning” OR “educational practice” OR synchronous OR asynchronous OR university OR “third-level education”
	(online OR internet OR web OR cyber*OR “distance learning” OR “blended learning” OR video OR telepresence OR digital OR virtual OR augmented OR computer OR 3D OR sim OR gamification OR “serious gam*”) AND (simulation)
**Strings 1, 2, 3 will be combined to identify articles that will undergo title and abstract screening**	

## Aim and objectives

The aim of this scoping review protocol is to explore the evidence reporting the use of inclusive pedagogy in online SBL in undergraduate nursing education. The following objectives will be addressed:

- To systematically identify and map the evidence to date regarding the use of inclusive pedagogy in online simulation-based learning in nursing education.
- To examine the degree to which the four components of inclusive pedagogy are developed in online SBL
- To explore the beliefs, theoretical frameworks, knowledge and practice which underpin nurse educators’ conceptualisations and development of inclusive pedagogy in online SBL.
- To identify the enablers and barriers of inclusive pedagogy in online simulation-based learning in undergraduate nursing education.
- To explore nursing students’ perceptions and experience of inclusive pedagogy and its use in online SBL.

### Stage 2: Identifying relevant studies

A three-step search strategy will be utilised in the scoping review ( Peters *et al.*, 2020); an initial limited search, a full search, and a manual screening of the reference lists of all the included articles will be completed by the author team. Both published and unpublished literature will be included in the scoping review.. The search strategy was developed using a combination of free text words, keywords and Medical Subject Headings (MeSH) and included Boolean operators (see Search Strategy, Table 1). The initial limited search will be undertaken in the Applied Science and Technology and CINAHL databases. From this search, the keywords will be analysed. A second search in all databases will be conducted using all identified keywords and index terms identified in the first search. An expert university librarian will be part of the research team to help refine the search strategy. Additional keywords and search terms will be added to the search strategy as they arise and will be documented accordingly and added to all other search strategies for other databases ( Page *et al.*, 2021). The databases were carefully selected from their subject areas to capture a broad search and include:

1. Applied Science and Technology Full Text (H.W. Wilson/EBSCO) (Computer Science)
2. Cumulative Index to Nursing and Allied Health Literature (CINAHL) (EBSCO) (Nursing)
3. ERIC (Educational Resources Information Center) International (Proquest) (Higher Education)
4. Pubmed (Nursing)
5. APA PsycInfo (Nursing)

Relevant grey literature such as exploratory studies, discussion papers, conference proceedings, graduate theses/dissertations will be included as described in Table 2.

**Table 2.** Grey literature sources.

Source	Search Terms
Google www.google.com	educator, inclusive design, nursing, online, simulation-based learning, undergraduate student (limited to the first 10 pages between years 2010–2022)
Open Grey http://www.opengrey.eu/	educator, inclusive design, nursing, online, simulation-based learning, undergraduate student (limited to the first 10 pages; between years 2010–2022)
Inclusive Design Research Centre https://achecker.ca/	educator, inclusive design, online simulation-based learning
The International Nursing Association for Clinical Simulation and Learning (INACSL) ( https://www.inacsl.org/)	educator, inclusive design, online simulation-based learning
Simulation Innovation Resource Center (SIRC) https://www.nln.org/education/education/sirc/sirc/sirc	educator, inclusive design, online simulation-based learning

### Stage 3: Study selection

Inclusion and exclusion criteria and rationale are summarised in Table 3. Each search conducted will be systematically documented (date, search terms, results per string) and saved by two independent authors. All eligible articles will be uploaded to Endnote X9 to remove duplicates before being imported into the systematic review platform, Covidence ( www.covidence.org). Reviewers will work independently during each stage of the reviewing process whereby all articles will be approved for inclusion by two authors. A pilot test of title and abstract screening (n=10) will be carried out by two reviewers to ensure consistency in inclusion criteria before screening the full article list. Once articles have been screened by title and abstract, the full texts of all articles will be reviewed by two reviewers. During both screening steps, where non-consensus occurs between the reviewers, a third reviewer will be asked to make the final decision. The results of the screening process will be detailed in a PRISMA-ScR flow diagram ( Page *et al.*, 2021; Tricco *et al.*, 2018).

**Table 3.** Inclusion and Exclusion Criteria.

Inclusion Criteria	Exclusion Criteria	Rationale
Studies on any of the four components of inclusive pedagogy: beliefs, knowledge, design and actions in the context of online SBL in undergraduate nursing education	Studies on inclusive pedagogy in any other context of nursing education including postgraduate nursing education Studies outside of nursing education.	Aligns with the research question and corresponding aims and objectives. Only studies related to the PCC will be included and therefore will aim to answer the research question.
Studies on online SBL in undergraduate nursing education	Studies on in-person SBL in undergraduate nursing education	Online SBL is the context of this review.
All primary peer-reviewed papers will be included.	Text and opinion papers will be excluded	The inclusion of peer reviewed papers on this topic will ensure the quality of published work.
Grey literature sources to include exploratory studies, discussion papers, conference proceedings and graduate theses/dissertations will be included	Any other type of paper will be excluded	To capture a broad overview of the research topic and in keeping with a realistic timeframe.
The literature search will be limited to between 1st January 2010 to 20th May 2022	Any papers published prior to 1st January 2010	Online simulation in nursing education only started to emerge in the early 2000’s, with significant technological advancements over the last 10 years. Therefore, the time period for this scoping review will be from 2010 to 2022.
Papers written in or translated to English language only	Papers written in a language other than English	The search will be limited to the English language only due to limited resources for translation.

### Stage 4: Data charting

A preliminary data extraction table has been created, using Microsoft Excel, by the author team to extract the key information to answer the review’s research questions (see Table 4). During data charting, two reviewers will pilot a small sample (n=3) of studies to ensure consistency and transparency in their extraction approach. Any non-consensus during this pilot test will be assessed by a third reviewer. This data extraction table may be further refined following the reading of identified studies to include other key information that may be important for the review.

**Table 4.** Sample Data Charting Table.

Chart Heading	Description
Author(s)	Name of author(s)
Year of publication	Year that the paper was published
Title of article	Title of the article or paper
Publication Type	The type of study or paper e.g. type of qualitative/quantitative/mixed methods etc.
Author Bibliometrics	How often has the research been cited?
Source /country of origin	The country where the study/review was conducted
Aims/purpose of research	The aim of the study
Study population	The population that was under investigation e.g., first year nursing students
Sample size	The sample size of the study
Study Methodology	What approach was used to investigate the research topic
Study Setting	e.g., Clinical site/university
Simulation Scenario - Nursing focus?	Simulation title and nursing focus
Theoretical or conceptual framework	Did the authors use a theoretical or conceptual framework in their study?
Data Collection Method	How was data collected? e.g., questionnaires/interviews
Data analysis Process	How was the data analysed?
Main Findings	What were the main findings of the study?
Conclusions	Important information reiterated in the conclusion
Definition of inclusive pedagogy	Is the term “inclusive pedagogy” defined in the study?
Authors cited for definition of ‘inclusive pedagogy’	What authors are commonly cited for the definition of ‘inclusive pedagogy’ in the literature to date?
Did the study address any of the components of inclusive pedagogy with regards to inclusion (below)?	Yes/No
Beliefs	Did the authors focus on educators’ belief systems regarding inclusion of students?
Knowledge	Did the authors focus on the knowledge required regarding the inclusion of students?
Design	Did the authors focus on the design of any type of online SBL regarding the inclusion of students?
Actions	What action(s) were conducted in any type of online SBL regarding the inclusion of students?
Enablers of inclusive pedagogy in online simulation-based learning in nursing education?	Were there any enablers to inclusive pedagogy identified in the study?
Barriers of inclusive pedagogy in online simulation-based learning in nursing education?	Were there any barriers to inclusive pedagogy identified in the study?
Implications of the research	What recommendations were made as a result of the research (i.e. education, practice, policy or future research)?
**Notes**	

### Stage 5: Collating, summarising and reporting the results.

During this stage, there will be regular team meetings organised by the first reviewer (LL) to discuss the analysis of the extracted data. The results along with the key findings will be organised in a descriptive narrative format includings figures and tables for quantitative data with reference to the research questions and objectives when reporting the results. The information analysed will include the frequency counts of concepts, populations and characteristics etc. and will clarify what is (un)known about inclusive pedagogy in online SBL in undergraduate nursing education and will subsequently identify gaps in the literature ( Peters *et al.*, 2020).

### Stage 6: Consultation and dissemination

The findings of this scoping review will inform educators on how inclusive pedagogy is presently visible in online SBL in undergraduate nursing education. Initially, the findings of this scoping review will be presented to key stakeholders in relevant fields; the EDI group of the HE Institutions, the clinical sites where the nursing students are linked to, as well as faculty and clinical partners involved in the teaching of nursing students and/or the development of online SBL programmes.

## Study status

The scoping review protocol is in stage 1, that includes the piloting and updating of search terms.

## Conclusion

Online SBL is a growing pedagogy in nursing education and research ( Jackson *et al.*, 2022; Stenseth *et al.*, 2022). In response to national policies, nurse educators are embedding EDI principles across curricula and seek strategies to ensure access and participation of all their students. However, to the best of the authors’ knowledge, no review exists which investigates the use of inclusive pedagogy in online SBL within undergraduate nursing education. This paper describes a review protocol that seeks to synthesize the literature on this topic. It will expose what is (un)known and will identify any existing gaps in the literature This review will have benefits for both informing and improving nursing education for both educators and students, as well as clinical practice. The results of the scoping review will be published in a peer-reviewed journal and presented at local, regional, national and international conferences.

## Data availability

### Underlying data

No data are associated with this article.

### Reporting guidelines

Figshare: The PRISMA-P Checklist for ‘Inclusive pedagogy in online simulation-based learning in undergraduate nursing education: A scoping review protocol', https://doi.org/10.6084/m9.figshare.19719262.v1. ( Langan *et al.*, 2022).

Data are available under the terms of the Creative Commons Attribution 4.0 International license (CC-BY 4.0).
