# Supplementary material for: Inclusive pedagogy in online simulation-based learning in undergraduate nursing education: A scoping review protocol
Source: HRB Open Res. 2022 Aug 15;5:39. Originally published 2022 May 16. [Version 2] doi: 10.12688/hrbopenres.13557.2 (PMC10170181; doi:10.12688/hrbopenres.13557.2)
Supplement: Peer review history

# Peer review: Reviewer response for version 2

**DOI:** 10.21956/hrbopenres.14870.r33719

**Published:** 2023-05-09

**Authors:** Ashley Franklin

**Affiliations:** 1 Harris College of Nursing & Health Sciences, Texas Christian University, Fort Worth, Texas, USA

Thank you for the opportunity to review this protocol. I appreciate the modifications you made based on an earlier peer review.  Your protocol is well-written, and the steps outlined follow Arksey and O'Malley's methods well.

 In the database search plan, I wonder about including EMBASE.

 I am very involved with INACSL and appreciate mention in Table 2.  I think rather than searching inacsl.org you may instead find it fruitful to search INACSL education programs and references from the INACSL Simulation Education Program (ISEP).  Note that these references are not public, rather there is a fee to "take the course." You'll find a similar model with the NLN Simulation Innovation Resource Center.  Alternatives that are available for free may include https://collaborate.uw.edu/online-training-and-resources/teaching-with-simulation-lessons/ or https://www.coursera.org/learn/clinicalsimulations

 If you are interested in locating dissertations, a common database used by graduate students in the US is Proquest Theses and Dissertations. Another is Open Access Theses and Dissertations.

 In terms of inclusion and exclusion criteria, I wonder if you would consider reviewing reference lists from scoping and/or systematic reviews in order to inform hand searches. You might also consider including studies published beyond English that would meet criteria in order to capture how many of those fit your PCC. This might enable future work to explore the topic of inclusivity in nursing simulation within the context of XYZ culture. By excluding them outright, you could miss an opportunity to recognize someone from a non-English speaking country already has a niche in this area.

Is the study design appropriate for the research question?

Yes

Is the rationale for, and objectives of, the study clearly described?

Yes

Are sufficient details of the methods provided to allow replication by others?

Yes

Are the datasets clearly presented in a useable and accessible format?

Not applicable

Reviewer Expertise:

Simulation in pre-licensure nursing education, simulation faculty development

I confirm that I have read this submission and believe that I have an appropriate level of expertise to confirm that it is of an acceptable scientific standard.

# Peer review: Reviewer response for version 1

**DOI:** 10.21956/hrbopenres.14803.r32330

**Published:** 2022-07-19

**Authors:** Rachel Macdiarmid

**Affiliations:** 1 Department of Nursing, Faculty of Health and Environmental Sciences, Auckland University of Technology, Auckland, New Zealand

Thank you for the opportunity to review your article. This article presents a scoping review protocol that aims to review the published and grey literature on inclusive pedagogy in online simulation-based learning in undergraduate nursing education. Overall, the protocol is well written and addresses an important issue in the current global context of nursing education.

**Introduction:**

 This is well structured and uses a clear framework to develop the background. I have a few suggestions for this section (see below). Nursing education and SBL is integrated well, and you develop a sound argument for the need for this scoping review.

 The sentence starting “As this concept offers” needs further clarity as I wondered which concept you were referring to specifically. In another paragraph you mention the third component “inclusive design” – I suggest distinguishing the first and second components.

**Methods:**

 The methods are well presented and identify the scoping review steps succinctly. The use of Arksey and O’Malley’s framework alongside the JBI guidelines and PRISMA-P demonstrates credibility and a clear structure.

**Design:**

 The research question has been appropriately developed using the PCC framework. The aims and objectives are clearly identified. Inclusion and exclusion criteria are stipulated and clarified with rationale. The types of evidence are clearly delineated. In regard to the search strategy, I wondered whether the terms simulation and simulation-based education or SBE need to be added to the context search terms. The databases identified are appropriate. The explanation of how the studies will be selected and reviewed is very clear and follows Peters *et al*., (2020) process. Collation, summarising, and reporting of results is well written and broad enough so that it responds to the aim of the review.

Is the study design appropriate for the research question?

Yes

Is the rationale for, and objectives of, the study clearly described?

Yes

Are sufficient details of the methods provided to allow replication by others?

Yes

Are the datasets clearly presented in a useable and accessible format?

Not applicable

Reviewer Expertise:

Nursing education; graduate nursing education; simulation based education; debriefing following simulation

I confirm that I have read this submission and believe that I have an appropriate level of expertise to confirm that it is of an acceptable scientific standard.

# Response

**Published:** 2022-08-10

**Authors:** Lisa Langan

Thank you very much for taking the time to review this protocol, and for your positive and valuable feedback. 

 1. Introduction:

 The introduction section has been revised to clarify and explain each of the four components of inclusive pedagogy. The concept of “inclusive design” has been distinguished from the concept of inclusive pedagogy in the amended version:

*Inclusive pedagogy* extends beyond teaching practices to the rationale behind how and why they are used ( Florian & Black-Hawkins, 2011). Moriña (2020), encompasses four specific components in her framework of inclusive pedagogy: *beliefs, knowledge, design and actions, *which could be used to guide educators in HE and thus will be the focus of this review. The first component *beliefs:* begins with a view that students’ contribution is valuable to their own learning and to the learning of others (Moriña, 2020). **Beliefs play a pivotal role in how educators develop their inclusive approaches. This can vary from providing for 1) needs common to all or 2) needs specific to sub-groups (e.g., an identified minority group) or 3) individual needs ( Stentiford & Koutsouris, 2021). The second component encapsulates the vast body of *knowledge *required by educators regarding various T&L strategies, how to support students and their needs, how and what students learn, and how to identify and evaluate student learning (Moriña, 2020). Florian and Black-Hawkins (2011) go further and note that the “craft of knowledge” required by educators must also include the iterative process of reflection and problem solving as these shape what is carried out in teaching practice.

 The third component, the *design *refers to the design of a pedagogy that recognizes the value of all students, involves them in the design of their studies from the beginning and meets the needs of as many students as possible ( Gale & Mills, 2013; Moriña, 2020). The third component of Moriña’s (2020) inclusive pedagogy framework share the same ideals as the Inclusive Design Research Centre’s (IDRC) portrayal of the concept of “Inclusive Design”, whereby individuals are involved throughout the design process from the beginning (IDRC, 2022). Inclusive Design is a prime example of an equity/equality, diversity and inclusion (EDI) theoretical framework,  underpinning accessibility and participation to the widest extent feasible ( Eikhaug & Gheerawo, 2021). Zhang *et al.* (2017:4) define inclusive design as: “ *the methodology of co-designing technological solutions with user groups to personalise their learning …to best support each individual*”.

 2. Design:

 The keywords “Simulation-based education” and “SBE” are added to the search strategy for each database.
